# Development of a Neutralization Assay for Influenza Virus Using an Endpoint Assessment Based on Quantitative Reverse-Transcription PCR

**DOI:** 10.1371/journal.pone.0056023

**Published:** 2013-02-20

**Authors:** Belete Teferedegne, Andrew M. Lewis, Keith Peden, Haruhiko Murata

**Affiliations:** Laboratory of DNA Viruses, Division of Viral Products, Office of Vaccines Research and Review, Center for Biologics Evaluation and Research, Food and Drug Administration, Bethesda, Maryland, United States of America; Pasteur Institute of Shanghai, Chinese Academy of Science, China

## Abstract

A microneutralization assay using an ELISA-based endpoint assessment (ELISA-MN) is widely used to measure the serological response to influenza virus infection and vaccination. We have developed an alternative microneutralization assay for influenza virus using a quantitative reverse transcription PCR-based endpoint assessment (qPCR-MN) in order to improve upon technical limitations associated with ELISA-MN. For qPCR-MN, infected MDCK-London cells in 96-well cell-culture plates are processed with minimal steps such that resulting samples are amenable to high-throughput analysis by downstream one-step quantitative reverse transcription PCR (qRT-PCR; SYBR Green chemistry with primers targeting a conserved region of the M1 gene of influenza A viruses). The growth curves of three recent vaccine strains demonstrated that the qRT-PCR signal detected at 6 hours post-infection reflected an amplification of at least 100-fold over input. Using ferret antisera, we have established the feasibility of measuring virus neutralization at 6 hours post-infection, a duration likely confined to a single virus-replication cycle. The neutralization titer for qPCR-MN was defined as the highest reciprocal serum dilution necessary to achieve a 90% inhibition of the qRT-PCR signal; this endpoint was found to be in agreement with ELISA-MN using the same critical reagents in each assay. qPCR-MN was robust with respect to assay duration (6 hours *vs*. 12 hours). In addition, qPCR-MN appeared to be compliant with the Percentage Law (*i.e.*, virus neutralization results appear to be consistent over an input virus dose ranging from 500 to 12,000 TCID_50_). Compared with ELISA-MN, qPCR-MN might have inherent properties conducive to reducing intra- and inter-laboratory variability while affording suitability for automation and high-throughput uses. Finally, our qRT-PCR-based approach may be broadly applicable to the development of neutralization assays for a wide variety of viruses.

## Introduction

Influenza serological studies are performed frequently in the context of clinical diagnosis for identification of human infection, global surveillance for circulating and emerging virus strains, virus antigenic characterization for seasonal vaccine-strain selection, and immunogenicity evaluation for vaccine licensure [1]. The hemagglutination-inhibition (HI) assay presently remains in wide use, in part owing to its relative simplicity and the existence of clinical data in support of establishing a serological correlate of protection against infection (a serum HI titer of at least 40 is generally considered to provide meaningful protection) [2], [3]. However, virus neutralization assays are increasingly being used to address shortcomings associated with the HI assay. For example, the source of red blood cells can often impact HI assay results; recent H3N2 viruses often exhibit reduced ability to hemagglutinate turkey red blood cells, thereby necessitating the use of red blood cells from an alternative source such as the guinea pig [4]. In some cases, the viral neuraminidase can contribute to hemagglutination activity apart from the conventional role associated with the viral hemagglutinin, thus complicating the interpretation of HI assay results [5]. Microneutralization (MN) assays for influenza virus using Madin-Darby canine kidney (MDCK) cells are often used as adjuncts or alternatives to the HI assay. In particular, a MN assay with an ELISA-based endpoint assessment has been broadly used due to the availability of a detailed protocol as well as associated reagents [6], [7]. Results obtained with MN assays have been shown to correlate with HI results [8]. However, MN assays appear to exhibit higher sensitivity for the detection of low-titer seroconversions [9]. The higher sensitivity of MN assays may be particularly useful in the assessment of human antibodies against avian influenza viruses [10]. Despite their advantages, MN assays appear to be associated with significantly greater inter- and intra-laboratory variability compared with the HI assay [11], [12], [13].

Despite the obvious technical advantages associated with quantitative PCR (qPCR) in terms of sensitivity and dynamic range, few studies have exploited this methodology to measure virus neutralization in a sero-epidemiological context [14], [15], [16], [17], [18]. The lack of widespread use of qPCR may be due, in part, to the fact that nucleic acid extraction from samples can be labor-intensive and rate-limiting. We recently developed a neutralization assay incorporating a SYBR Green qPCR endpoint assessment for SV40, a DNA virus of the polyomavirus family [19]. We demonstrated that crude virus samples can be used directly as amplification templates in qPCR without nucleic acid extraction, provided that they undergo sufficient dilution to eliminate the impact of PCR inhibitors. A similar high-throughput approach that obviates the need for nucleic acid extraction might be feasible for other DNA viruses.

We wished to extend our streamlined qPCR approach to encompass RNA viruses. RNA as a PCR target, as opposed to DNA, poses additional obstacles. Nucleases present in samples that contribute to the lability of RNA must be mitigated. Additionally, one must contend with a reverse transcription step to convert the target RNA to cDNA prior to proceeding with downstream qPCR.

In the present study, we demonstrate the feasibility of using a qPCR-based assessment for measuring the neutralization of an RNA virus such as influenza virus. We have emphasized an approach that minimizes sample manipulation steps so as to facilitate throughput. We make use of a commercially available reagent that allows the straightforward preparation of sample lysates from infected cells that are amenable to downstream one-step quantitative reverse transcription PCR (qRT-PCR) without the need for RNA extraction. For qRT-PCR, we routinely rely on an automated liquid handling system to prepare reactions, thereby improving throughput and data quality. The sensitivity of qPCR allows the assessment of endpoint within a single virus replication cycle. In addition, the dynamic range of qPCR allows our neutralization assay to be robust to perturbations in input virus dose. Thus, our qPCR-based assay might have inherent properties conducive to reducing intra- and inter-laboratory variability associated with existing MN assays for influenza virus while affording suitability for automation and high-throughput applications.

## Materials and Methods

### Ethics Statement

Ethics approval by the Research Involving Human Subjects Committee (RIHSC) at the US Food and Drug Administration was obtained for the use of human sera. The sera were obtained with written informed consent from all individuals.

### Cell Culture

MDCK-London cells [7] at passage 12 were obtained from J. Weir (Division of Viral Products, CBER, FDA; Bethesda, MD). Cells were grown in DMEM (15-013-CV; Mediatech, Inc.) supplemented with 10% fetal bovine serum (Hyclone) and 2 mM glutamine. For all experiments, cells were used prior to passage 30.

### Viruses and Sera for Neutralization Experiments

The following viruses (propagated in embryonated chicken eggs) were obtained from V. Lugovtsev and J. Weir (Division of Viral Products, CBER, FDA): IVR-148, which was derived from A/Brisbane/59/2007 (H1N1); IVR-145, which was derived from A/Solomon Islands/3/2006 (H1N1); and NYMC X-175C, which was derived from A/Uruguay/716/2007 (H3N2). These are reassortant viruses used in recent seasonal vaccine formulations. Working virus stocks for our experiments, generated after a single passage in MDCK-London cells, were aliquoted and stored frozen at −80°C prior to use.

Corresponding ferret sera (prepared from intranasally infected animals bled after three weeks and treated with receptor destroying enzyme) were also obtained from V. Lugovtsev and J. Weir. The hemagglutination-inhibition titer was 1024 against the homologous virus for each of the three sera (V. Lugovtsev, personal communication).

Adult human sera, described in Wang *et al*. [20], were acquired from W. Wang and C. Weiss (Division of Viral Products, CBER, FDA). These were collected in September-December of 2009 from volunteers who had received recent seasonal influenza vaccines (2004/05 to 2008/09). The samples were heat-inactivated by incubation at 56°C for 30 minutes prior to use in neutralization assays.

### ELISA-based Influenza Virus Microneutralization Assay (ELISA-MN)

A microneutralization assay for influenza virus with an ELISA-based endpoint assessment was performed as described by a protocol available from the World Health Organization (WHO) [6] with minor modifications. Instead of 96-well plates specifically designed for ELISA as recommended by the WHO protocol (for example, Immulon-2HB available from Thermo Scientific), we used flat-bottom plates designed for cell culture (Nunc MicroWell Plates; catalog number 167008 distributed by Thermo Scientific) because (1) we experienced difficulty with efficient cell adherence when using ELISA plates especially in the presence of exogenous trypsin, and (2) we reasoned that the acetone fixation step would immobilize antigens for downstream ELISA, and thus, the high-binding surface attribute of ELISA plates may not be necessary. Other critical reagents were obtained from sources recommended by the WHO protocol: RIA grade bovine serum albumin (A7888; Sigma; St. Louis, MO), TPCK-trypsin (T1426; Sigma), o-phenylenediamine dihydrochloride (P8287; Sigma), monoclonal anti-influenza A virus nucleoprotein (NP) clones A1 and A3 ascites blend (NR-4282; BEI Resources; Manassas, VA), and goat anti-mouse IgG conjugated to horse radish peroxidase (074–1802; KPL, Inc.; Gaithersburg, MD).

Virus stocks were titrated with MDCK-London cells as described in the WHO protocol in the presence or absence of TPCK-trypsin (1 µg/mL) using ELISA to identify culture wells positive for virus growth; the TCID_50_ for each stock was calculated by the method of Reed-Muench [21]. To perform ELISA-MN, infection was performed in DMEM supplemented with glutamine (2 mM), HEPES (25 mM), and bovine serum albumin (0.2%); TPCK-trypsin (1 µg/mL) was also included where appropriate. Virus inoculum (100 TCID_50_, as calculated by ELISA-based titration, in 50 µL) was mixed with a dilution of serum to be tested (50 µL; from a 2-fold dilution series) in a well of a 96-well culture plate. Following an incubation for 1 h at 37°C, a suspension of MDCK-London cells (15,000 cells in 100 µL) was added per well. At 22 h post-infection, cells were fixed with cold 80% acetone in PBS, and virus growth in each well was assessed by ELISA as described in the WHO protocol. The neutralization titer was determined as the reciprocal of the highest serum dilution (prior to the addition of virus or cells) resulting in at least 50% inhibition of the ELISA signal.

### qRT-PCR-based Influenza Virus Microneutralization Assay (qPCR-MN)

The protocol for qPCR-MN was modeled after the WHO protocol for ELISA-MN. Virus stocks were titrated with MDCK-London cells at 72 h post-infection by microscopic observation of CPE. Virus inoculum (1000 TCID_50_, as calculated by CPE-based titration, in 50 µL) was mixed with a dilution of serum to be tested (50 µL) in a well of a 96-well culture plate. Following an incubation for 1 h at 37°C, a suspension of MDCK-London cells (30,000 cells in 100 µL) was added per well. At 6 h post-infection, virus growth in each well was quantified by qRT-PCR.

Lysates from MDCK-London cells grown in 96-well culture plates (in the presence or absence of influenza virus infection) were prepared using the iScript Sample Preparation Reagent (referred to as SPR; 170–8898; Bio-Rad; Hercules, CA) according to instructions provided by the supplier. Cells were washed once with 200 µL PBS, and 100 µL of SPR were added per well. After incubation for 2 min at room temperature (∼22°C), the resulting cell lysates were collected and stored frozen at −20°C until assessment by qRT-PCR. Remaining samples were re-frozen and stored at −20°C.

The primers for qRT-PCR (forward: AAGACCAATCCTGTCACCTCTGA; reverse: CAAAGCGTCTACGCTGCAGTCC) amplify a 104 bp product in a highly conserved region of the M1 gene of influenza A viruses [22]. These primers were originally intended to be used in conjunction with a Taqman probe; however, we chose to dispense with using a conjugated probe in favor of the simpler SYBR Green qPCR chemistry in order to minimize cost. Following optimization (performed essentially as described previously [19], in terms of primer concentration and annealing temperature), each reaction contained: template (1 µL of SPR lysate), 1X iScript One-Step SYBR Green RT-PCR Supermix (170–8893; Bio-Rad), 600 nM of each primer (synthesized at the Facility for Biotechnology Resources; CBER, FDA; Bethesda, MD), and nuclease-free water to 10 µL. Distribution of PCR cocktail (RT-PCR Supermix and primers) and experimental samples (SPR lysates) to 96-well PCR plates was routinely performed with an epMotion 5075 automated liquid handling system (Eppendorf North America; Hauppauge, NY). PCR data were analyzed using a CFX96 real-time PCR instrument (Bio-Rad) with the accompanying software. The thermocycling protocol was as follows: 50°C for 10 min (1X), 95°C for 5 min (1X), 95°C for 10 s/61°C for 15 s/72°C for 30 s (40X); data collection occurred after the 72°C extension step. Total RNA purified from MDCK-London cells infected with the influenza virus strain A/PR/8/34 (prepared with the RNeasy Kit; Qiagen USA; Valencia, CA) was used as a qRT-PCR quantification standard. In order to enhance comparability with experimental samples, the diluent used for the RNA standard was SPR lysate prepared from uninfected MDCK-London cells (the initial dilution contained 10 ng of standard RNA per µL). For each PCR plate, a 10-fold dilution series of the standard was assessed in at least duplicate. The quantity of RNA in each experimental sample was normalized against the mean value (n≥3) obtained from control wells in which cells were infected in the absence of neutralizing serum (virus control wells). The neutralization titer was determined as the reciprocal of the highest serum dilution (prior to the addition of virus or cells) resulting in at least 90% inhibition of the qRT-PCR signal.

For robustness assessments of qPCR-MN, the relevant assay parameters (input virus dose, assay duration ± TPCK-trypsin) were modulated while leaving others unaltered.

## Results

### Performance Analysis of SYBR-Green qRT-PCR

**Figure 1:**
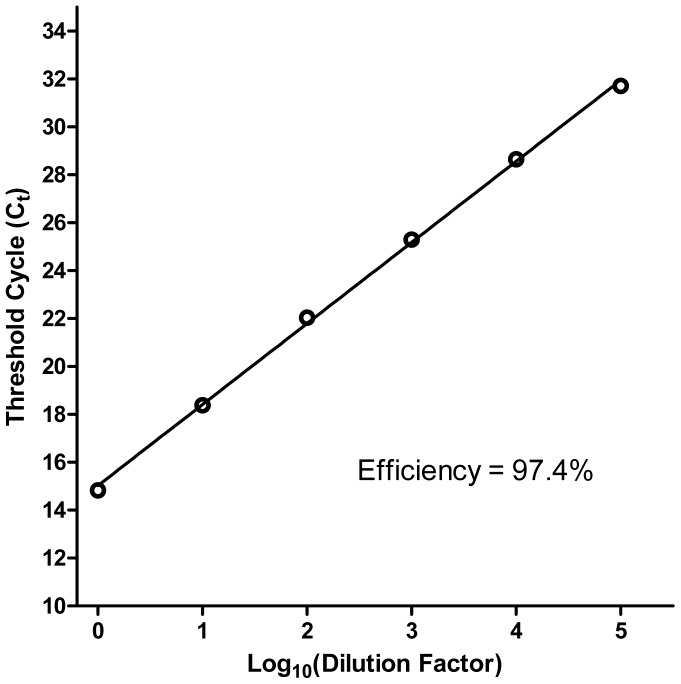
Threshold cycle (C_t_) *vs*. log_10_(dilution factor) from SYBR Green qRT-PCR (targeting the M1 gene of influenza A viruses) applied to a serial dilution of total RNA purified from MDCK-London cells infected with A/PR/8/34 (n = 3 for each dilution), which was used as an RNA quantification standard for our experiments. Lysate from uninfected MDCK-London cells prepared with the Bio-Rad iScript Sample Preparation Reagent (SPR) was used as the diluent to prepare the RNA serial dilution in order to achieve comparability with experimental samples. The initial dilution contained 10 ng of standard RNA per µL.

We have made use of a commercially available reagent, the Bio-Rad iScript Sample Preparation Reagent (referred to hereafter as SPR), to prepare cell lysates amenable for direct assessment by downstream quantitative reverse transcription PCR (qRT-PCR) with minimal attendant processing steps. Lysates from MDCK-London cells grown in 96-well culture plates (in the presence or absence of influenza virus infection) were prepared by washing the cells once with PBS and adding 100 µL of SPR per well. After incubation for 2 min at room temperature, the resulting cell lysates were collected and stored frozen at −20°C until assessment by qRT-PCR. In order to generate a quantification standard for qRT-PCR, total RNA was purified from MDCK-London cells infected with the influenza virus strain A/PR/8/34 and a dilution series was prepared using SPR lysate from uninfected MDCK-London cells as the diluent (in order to achieve comparability with experimental samples). The performance of our one-step SYBR Green qRT-PCR, using primers targeting a highly conserved region of the matrix gene of influenza A viruses [22], was verified by assessing a dilution series of the quantification standard. Typical results are shown in Fig. 1. The PCR efficiency was routinely 95–100%. Linearity was observed over at least a 5 log_10_ range. A dilution series of the RNA standard was assessed in each qRT-PCR run in order to facilitate the quantification of influenza virus RNA copies in experimental samples.

### Assessment of Influenza Virus Replication Kinetics by qRT-PCR

**Figure 2:**
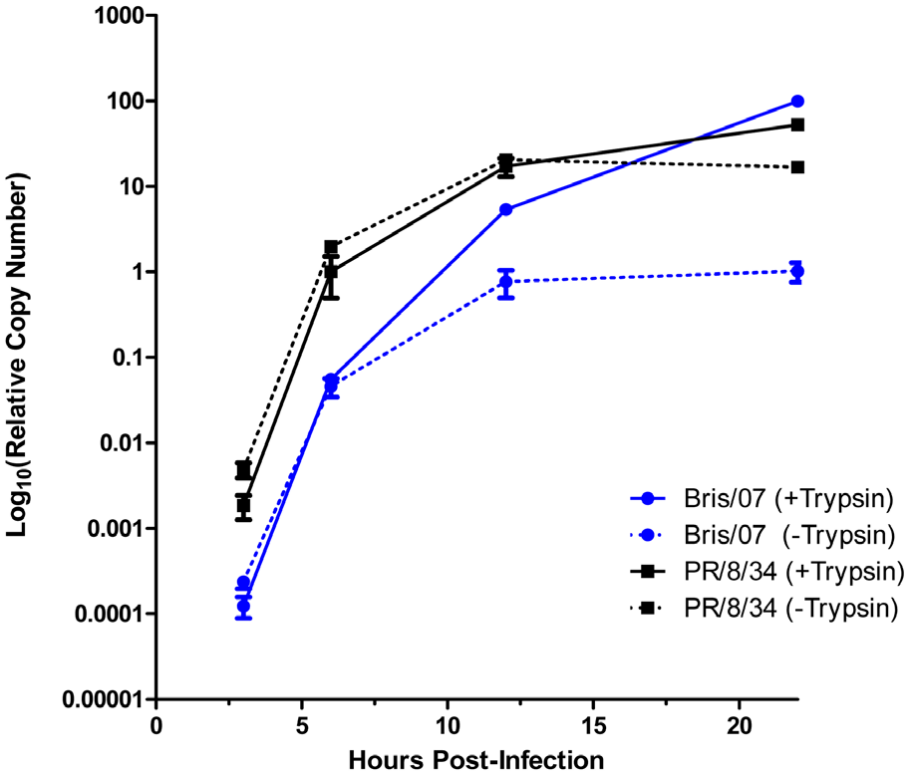
Replication kinetics of influenza virus assessed by qRT-PCR. Virus replication was assessed in the absence or presence of TPCK-trypsin (1 µg/mL). Virus (1000 TCID_50_ in 100 µL) was placed into a well of a 96-well plate. After incubation for 1 h at 37°C, a suspension of MDCK-London cells (30,000 in 100 µL) was added. At the indicated times, experimental samples were prepared using SPR and subjected to qRT-PCR. The RNA copy numbers were normalized to the mean value observed for A/PR/8/34 at 6 hours in the presence of TPCK-trypsin. Each point represents the mean ± SEM (n = 3).

Our qRT-PCR approach was used to assess the replication kinetics of influenza virus. Per well of a 96-well plate, an inoculum containing 1000 TCID_50_ of virus, either A/PR/8/34 or a vaccine reassortant derived from A/Brisbane/59/2007 (Bris/07), was mixed with 30,000 trypsinized MDCK-London cells in suspension in the absence or presence of 1 µg/mL TPCK-trypsin. At 3, 6, 12, and 22 hours post-infection, cell lysates were prepared using SPR and subjected to qRT-PCR. The results are shown in Fig. 2. The RNA copy numbers were normalized to the mean value observed for A/PR/8/34 at 6 hours in the presence of TPCK-trypsin. For Bris/07 in the absence of exogenous trypsin, the qRT-PCR signal reached a plateau by 12 hours post-infection; in the presence of exogenous trypsin, the qRT-PCR signal continued to increase throughout the duration of the experiment. Thus, following the successful infection of initial cells, subsequent rounds of infection by Bris/07 appear to require exogenous trypsin. Two other vaccine reassortant strains derived from A/Solomon Islands/03/2006 and A/Uruguay/716/2007 (SI/06 and Uru/07, respectively) behaved in a manner similar to Bris/07 (data not shown). In contrast, the growth curves for A/PR/8/34 in the absence or presence of trypsin were similar to each other. This underscores the strain dependence known to be associated with the requirement for trypsin to sustain efficient replication of influenza virus in cultured cells. Ample qRT-PCR signal was observed for both Bris/07 and A/PR/8/34 at 6 hours post-infection in the absence or presence of exogenous trypsin; the approximate range for the qPCR threshold cycles (C_t_) at this time was 23 to 25, within the quantifiable range for qRT-PCR by a comfortable margin. By comparing the qRT-PCR signal at 3 hours and 6 hours, one can estimate that the input was amplified by >100-fold (the addition of cells as a suspension precluded the direct assessment of input signal at 0 hours). The data support the feasibility of measuring the endpoint in a virus neutralization assay at 6 hours post-infection.

### Discrimination of 2-fold Variations in Virus Input by qRT-PCR

**Figure 3:**
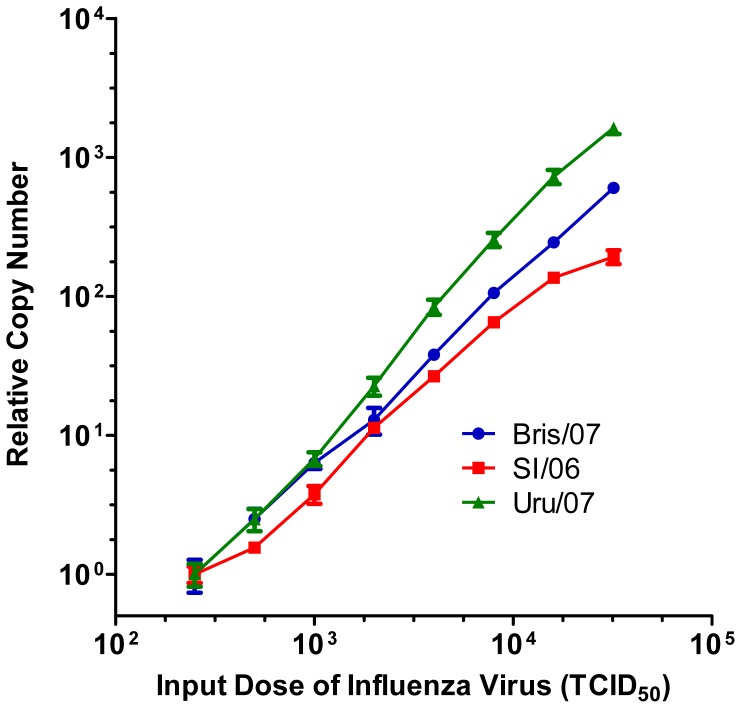
Discrimination of 2-fold variations in virus input. Virus replication was assessed in the presence of TPCK-trypsin (1 µg/mL). A 2-fold dilution series of the virus was prepared (32,000 to 250 TCID_50_ per 100 µL per well). After incubation for 1 h at 37°C, a suspension of MDCK-London cells (30,000 in 100 µL) was added. At 6 h post-infection, experimental samples were prepared using SPR and subjected to qRT-PCR. For each virus strain, the RNA copy numbers were normalized to the mean value observed from cells infected with 250 TCID_50_. Each point represents the mean ± SEM (n = 3).

We assessed the capability of our approach to discriminate 2-fold variations in virus input. Trypsinized MDCK-London cells (30,000 cells per well of a 96-well plate) were mixed with a dilution from a 2-fold dilution series of virus (Bris/07, SI/06, or Uru/07) ranging from 250 to 32,000 TCID_50_. Infection proceeded in the presence of 1 µg/mL TPCK-trypsin. At 6 hours post-infection, cell lysates were prepared using SPR and subjected to qRT-PCR. The results are shown in Fig. 3. For each virus strain, the RNA copy numbers were normalized to the mean value observed from cells infected with 250 TCID_50_. Reasonable linearity was observed for each virus strain. Thus, our qRT-PCR approach appears to be capable of discerning 2-fold differences in virus input over a wide range. The data support the feasibility of using qRT-PCR for endpoint assessment in the context of a virus neutralization assay in which the effective virus input is modulated by the presence of neutralizing antibodies.

### qRT-PCR-based Microneutralization Assay (qPCR-MN)

**Figure 4:**
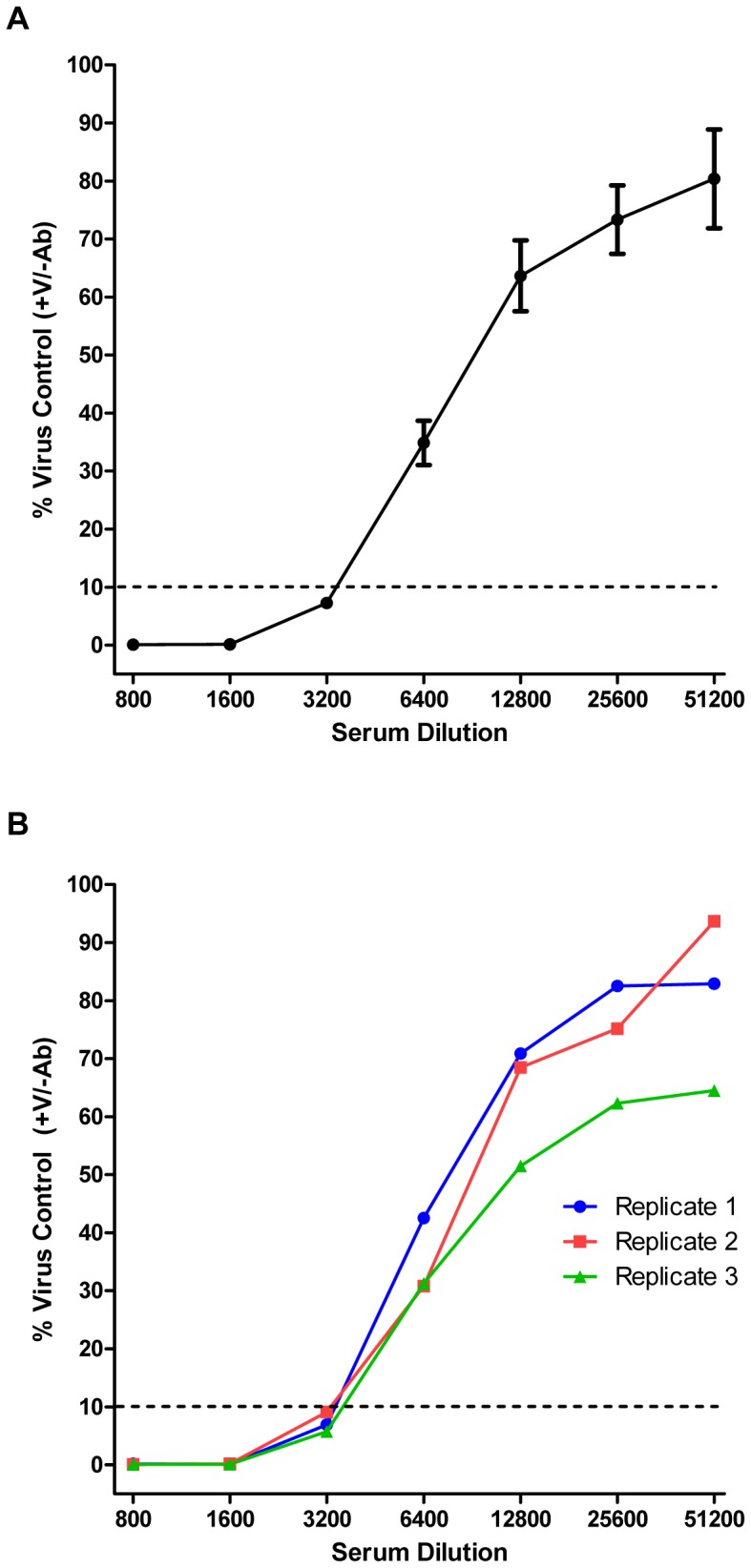
Influenza virus microneutralization assessed by qRT-PCR (qPCR-MN). (**A**) An inoculum containing 1000 TCID_50_ of virus (Bris/07) was mixed with a dilution from a 2-fold dilution series of ferret antiserum in a well of a 96-well plate. After allowing the neutralization reaction to proceed for 1 hour at 37°C, trypsinized MDCK-London cells (30,000 per well) were added. TPCK-trypsin was present at 1 µg/mL. After 6 hours, experimental samples were prepared using SPR and subjected to qRT-PCR. The RNA copy numbers were normalized to the mean value obtained from infected wells in the absence of neutralizing serum (virus control wells). Each point represents the mean ± SEM (n = 3). The neutralization titer was defined as the reciprocal of the highest dilution factor of serum necessary to inhibit the PCR signal by 90%. (**B**) Same data as in (A); however, each experimental replicate was assessed independently. The mean of these curves would result in the curve depicted in (A).

Virus neutralization was assessed by our qRT-PCR approach using ferret post-infection sera. An inoculum containing 1000 TCID_50_ of virus was mixed with a dilution from a 2-fold dilution series of antiserum in a well of a 96-well plate. After allowing the neutralization reaction to proceed for 1 hour at 37°C, trypsinized MDCK-London cells (30,000 per well) were added. TPCK-trypsin was present at 1 µg/mL. After 6 hours, cell lysates were prepared using SPR and subjected to qRT-PCR. A representative neutralization result using Bris/07 and its corresponding ferret antiserum is shown in Fig. 4A. The RNA copy numbers were normalized to the mean value obtained from infected wells in the absence of neutralizing serum (virus control wells). Each of the data points represents the mean of three experimental replicates. An inhibition of qRT-PCR signal was observed in a manner dependent on the concentration of neutralizing serum. The neutralization titer was defined as the reciprocal of the highest dilution factor of serum necessary to inhibit the PCR signal by 90%. Thus, the neutralization titer for Bris/07 serum against the homologous virus strain was 3200 according to this experiment.

Each of the three replicates associated with the experiment shown in Fig. 4A represents an independent serum dilution series (corresponding to a column of a 96-well plate), and thus, can be evaluated independently to derive an estimate of the neutralization titer. An example of such an analysis is demonstrated in Fig. 4B, which is derived from the same experiment (Bris/07 and its corresponding serum) as in Fig. 4A; the mean of the curves depicted in Fig. 4B would result in the curve shown in Fig. 4A. The neutralization titer (90% inhibition) determined for each of the three experimental replicates in Fig. 4B was 3200. Thus, our qRT-PCR-based microneutralization assay (qPCR-MN) appears to provide consistent estimates of the neutralization titer within an experiment.

qPCR-MN results (using three virus strains and their corresponding post-infection ferret sera) from four independent experiments, with three replicates for each experiment, are shown in Table 1. The geometric mean titer (GMT; n = 12) was 3020, 2690, and 2690 for Bris/07 serum, SI/06 serum, and Uru/07 serum, respectively, against the homologous virus. The GMT (Experiment 1; n = 3) was 1280 for Bris/07 serum against SI/06 virus, but <80 against Uru/07 virus; similarly, the GMT (Experiment 1; n = 3) was 254 for SI/06 serum against Bris/07 virus, but <80 against Uru/07 virus. Uru/07 serum demonstrated low neutralizing activity (<80; Experiment 1) against the other two viruses (Bris/07 and SI/06). The heterologous neutralization results are consistent with expectation considering the antigenic relationships of the three viruses (Bris/07 and SI/06 are H1N1 viruses, whereas Uru/07 is an H3N2 virus) and demonstrate the specificity of the reagents used in our experiments. Overall, qPCR-MN appears to provide consistent results both within and between experiments, with titers from experimental replicates differing by at most 2-fold.

**Table 1:** qPCR-MN titers determined for each experimental replicate.

	Virus	Bris/07 (B)			SI/06 (SI)			Uru/07 (U)		
	Serum	B	SI	U	B	SI	U	B	SI	U
**Exp 1**	**Replicate 1**	3200	320	<80	1280	1600	<80	<80	<80	1600
	**Replicate 2**	3200	160	<80	1280	1600	<80	<80	<80	1600
	**Replicate 3**	3200	320	<80	1280	1600	<80	<80	<80	1600
**Exp 2**	**Replicate 1**	1600	NA		NA	3200	NA	NA		3200
	**Replicate 2**	3200				3200				3200
	**Replicate 3**	3200				3200				3200
**Exp 3**	**Replicate 1**	3200				3200				3200
	**Replicate 2**	3200				3200				3200
	**Replicate 3**	3200				3200				3200
**Exp 4**	**Replicate 1**	3200				3200				3200
	**Replicate 2**	3200				3200				3200
	**Replicate 3**	3200				3200				3200
**GMT**		3020	254	<80	1280	2690	<80	<80	<80	2690

### ELISA-based Microneutralization Assay (ELISA-MN)

We wished to compare our qPCR-MN results with those obtained using the established ELISA-based microneutralization assay (ELISA-MN). Per protocol [6], ELISA-MN requires titration of virus stock infectivity by scoring the absence or presence of virus replication by ELISA. In contrast, for qPCR-MN, we relied on infectivity titration determined by microscopic observation of CPE. The virus stock infectivity assessment by ELISA (at 22 hours in the absence or presence of exogenous trypsin) or by scoring CPE (at 72 hours in the presence of exogenous trypsin) is summarized in Table 2. In general, the following relationship was observed:

**Table 2:** Infectivity titers for virus stocks determined by ELISA (at 22 hours, ±TPCK-trypsin) or microscopic observation of CPE (at 72 hours, +TPCK-trypsin).

Virus	TCID_50_/mL (CPE/+Trypsin/72Hours)	TCID_50_/mL (ELISA/+Trypsin/22Hours)	TCID_50_/mL (ELISA/NoTrypsin/22Hours)
**Bris/07**	1.9×10^8^	6.3×10^7^	1.9×10^6^
**SI/06**	5.6×10^8^	3.9×10^7^	1.9×10^6^
**Uru/07**	6.3×10^7^	5.6×10^6^	1.8×10^6^

TCID_50_(+Trypsin, CPE)>TCID_50_(+Trypsin, ELISA)>TCID_50_(−Trypsin, ELISA).

Per protocol, 100 TCID_50_ was the input virus dose for ELISA-MN, using values determined in the presence or absence of exogenous trypsin, as appropriate, according to the experimental condition used in ELISA-MN. It is important to note that compared with our standard virus input of 1000 TCID_50_(CPE) for qPCR-MN, the ELISA-MN virus input of 100 TCID_50_(ELISA) can be greater in absolute terms depending on the experimental condition used.

ELISA-MN was performed in the absence or presence of TPCK-trypsin. The endpoint was assessed at 22 hours post-infection. ELISA-MN results from three independent experiments (with each experiment consisting of three replicates) are shown in Table 3. ELISA-MN titer (per protocol, corresponding to the highest reciprocal dilution resulting in 50% inhibition of the ELISA signal) in the absence of exogenous trypsin was 3200 for each of the three sera against the homologous virus, in agreement with the qPCR-MN titers. ELISA-MN titer in the absence of trypsin was consistently 2-fold higher than the ELISA-MN titer in the presence of trypsin for the homologous neutralization of SI/06 and Uru/07. This is most likely due to the impact on the neutralization titer attributable to multiple cycles of virus replication allowed by the presence of trypsin.

**Table 3:** ELISA-MN titers determined in the absence or presence of TPCK-trypsin (n = 3 for each experiment).

Virus	Bris/07		SI/06		Uru/07	
Serum	Bris/07		SI/06		Uru/07	
TPCK-Trypsin	−	+	−	+	−	+
Exp 1	3200	1600	3200	1600	3200	1600
Exp 2	3200	3200	3200	1600	3200	1600
Exp 3	3200	3200	3200	1600	3200	1600

### qPCR-MN Robustness Assessment: Assay Duration (±TPCK-Trypsin)

An assessment of qPCR-MN robustness was performed with respect to assay duration. The parameters of qPCR-MN (1000 TCID_50_ virus input) were maintained as before except that neutralization was assessed in the presence or absence of TPCK-trypsin at 6, 12, and 22 hours post-infection. qPCR-MN results from three independent experiments, with three replicates for each experiment, are shown in Tables 4, 5, 6. qPCR-MN appears to be robust to perturbations in assay duration. Consistent titers were observed within and across experiments at 6 hours (±trypsin), 12 hours (±trypsin), and 22 hours (-trypsin). Titers at 22 hours in the presence of exogenous trypsin were 2-4-fold lower compared with the other experimental conditions, likely due to the effect of multiple cycles of virus infection (corroborating the ELISA-MN results described in Table 3).

**Table 4:** qPCR-MN robustness assessment with respect to assay duration (±TPCK-trypsin): Bris/07.

	Virus	Bris/07					
	Serum	Bris/07					
	Duration	6 h		12 h		22 h	
	TPCK-Trypsin	−	+	−	+	−	+
**Exp 1**	**Rep 1**	3200	3200	3200	3200	3200	3200
	**Rep 2**	3200	3200	3200	3200	3200	3200
	**Rep 3**	3200	3200	3200	3200	3200	3200
**Exp 2**	**Rep 1**	3200	3200	3200	3200	3200	1600
	**Rep 2**	3200	3200	3200	3200	3200	1600
	**Rep 3**	3200	3200	3200	3200	3200	1600
**Exp 3**	**Rep 1**	3200	3200	3200	3200	3200	1600
	**Rep 2**	3200	3200	3200	3200	3200	1600
	**Rep 3**	3200	3200	3200	3200	3200	1600

**Table 5:** qPCR-MN robustness assessment with respect to assay duration (±TPCK-trypsin): SI/06.

	Virus	SI/06					
	Serum	SI/06					
	Duration	6 h		12 h		22 h	
	TPCK-Trypsin	−	+	−	+	−	+
**Exp 1**	**Rep 1**	3200	3200	3200	3200	3200	1600
	**Rep 2**	3200	3200	3200	3200	3200	800
	**Rep 3**	3200	3200	3200	3200	3200	800
**Exp 2**	**Rep 1**	3200	3200	1600	1600	6400	1600
	**Rep 2**	3200	3200	1600	1600	3200	1600
	**Rep 3**	3200	3200	1600	1600	1600	1600
**Exp 3**	**Rep 1**	3200	3200	3200	3200	3200	1600
	**Rep 2**	3200	3200	3200	3200	1600	1600
	**Rep 3**	3200	3200	3200	3200	1600	1600

**Table 6:** qPCR-MN robustness assessment with respect to assay duration (±TPCK-trypsin): Uru/07.

	Virus	Uru/07					
	Serum	Uru/07					
	Duration	6 h		12 h		22 h	
	TPCK-Trypsin	−	+	−	+	−	+
**Exp 1**	**Rep 1**	6400	3200	3200	3200	3200	1600
	**Rep 2**	3200	3200	3200	3200	3200	1600
	**Rep 3**	3200	3200	3200	3200	3200	1600
**Exp 2**	**Rep 1**	3200	3200	3200	3200	3200	1600
	**Rep 2**	3200	3200	3200	3200	3200	1600
	**Rep 3**	3200	3200	3200	3200	3200	1600
**Exp 3**	**Rep 1**	3200	6400	3200	3200	3200	800
	**Rep 2**	3200	3200	3200	3200	3200	1600
	**Rep 3**	3200	3200	3200	3200	3200	1600

### qPCR-MN Robustness Assessment: Input Virus Dose

An assessment of qPCR-MN robustness was also performed with respect to input virus dose. Neutralization assays were allowed to proceed for 6 hours in the presence of TPCK-trypsin; the input virus dose was 500, 1000, 3000, or 12,000 TCID_50_. qPCR-MN results from two independent experiments, with three replicates for each experiment, are shown in Table 7. Overall, qPCR-MN appears to be robust across a >10-fold range of input virus; however, a 2-fold reduction in titer (3200→1600) was observed at the highest input virus (12,000 TCID_50_) for one of the two experiments with SI/06.

**Table 7:** qPCR-MN robustness assessment with respect to input virus dose.

	Virus	Bris/07				SI/06				Uru/07			
	Serum	Bris/07				SI/06				Uru/07			
	Input Virus (TCID_50_)	500	1000	3000	12000	500	1000	3000	12000	500	1000	3000	12000
**Exp 1**	**Rep 1**	3200	3200	3200	3200	3200	3200	3200	3200	3200	3200	3200	3200
	**Rep 2**	3200	3200	3200	3200	3200	3200	3200	3200	3200	3200	3200	3200
	**Rep 3**	3200	3200	3200	3200	3200	3200	3200	3200	3200	3200	3200	3200
**Exp 2**	**Rep 1**	3200	3200	3200	3200	3200	3200	3200	1600	1600	3200	3200	3200
	**Rep 2**	3200	3200	3200	3200	3200	3200	3200	1600	3200	3200	3200	1600
	**Rep 3**	1600	1600	3200	3200	3200	3200	3200	1600	3200	3200	3200	3200

### Correlation between Neutralization Titers Measured by qPCR-MN and ELISA-MN

**Figure 5:**
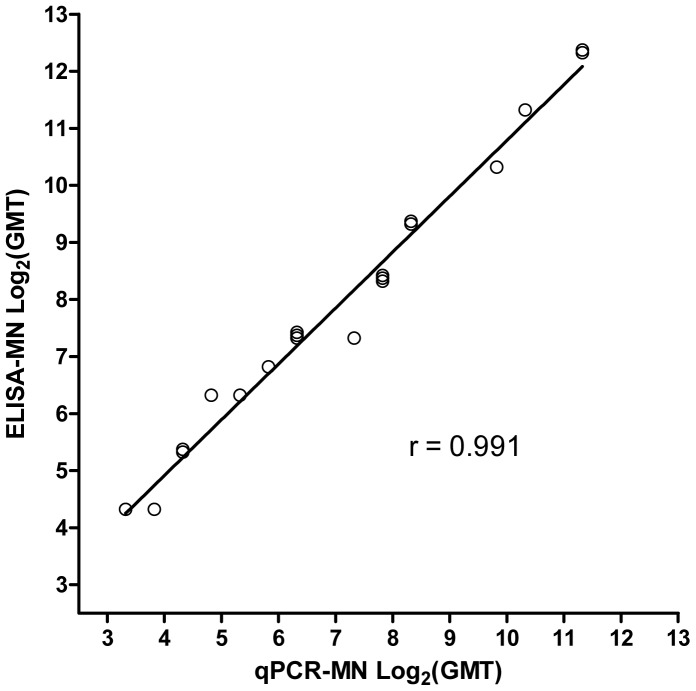
Correlation between neutralization titers measured by qPCR-MN and ELISA-MN. Adult human sera (n = 20) were assessed by qPCR-MN (input virus of 1000 TCID_50_ scored by CPE; endpoint assessment at 6 hours post-infection; +TPCK-trypsin) and ELISA-MN (input virus of 100 TCID_50_ scored by ELISA; endpoint assessment at 22 hours post-infection; -TPCK-trypsin). Neutralization activity against SI/06 was measured. Each point represents the log_2_ of the geometric mean titer derived from two experimental replicates. Certain points were nudged along the y-axis direction in order to reveal overlaps.

Adult human serum samples (n = 20; characterized in a study by Wang *et al*. [20]) from individuals who had received recent seasonal influenza vaccines (2004/05 to 2008/09) were assessed by qPCR-MN and ELISA-MN. Neutralization activity against SI/06 was measured. The results are shown in Fig. 5. Both assays yielded similar titers (≤2-fold difference for 19 out of 20 samples; <3-fold difference for the remaining sample). The correlation coefficient was 0.991. For ELISA-MN, a neutralization titer of 160 in adults has been proposed as a possible threshold signifying protection against infection by influenza virus [23]. The correlation in results obtained by qPCR-MN and ELISA-MN was maintained throughout the range of titers tested, both above and below the threshold of 160 (10 out of 20 samples had titers <160 as measured by qPCR-MN; 7 out of 20 samples had titers <160 as measured by ELISA-MN). The performance of qPCR-MN with low-activity samples suggests that its sensitivity for detecting neutralization activity is comparable with ELISA-MN.

## Discussion

We have successfully demonstrated the feasibility of using qRT-PCR to quantify the extent of influenza virus neutralization. RNA extraction from experimental samples, heretofore a significant rate-limiting step, was circumvented by making use of a commercially available reagent that allows the generation of cell lysates suitable as input for downstream qRT-PCR. We have found that consistent qRT-PCR results can be obtained from replicate samples prepared in this straightforward manner. The procedure for our qRT-PCR- based microneutralization assay (qPCR-MN) was modeled after the established ELISA-based microneutralization assay (ELISA-MN) [6] and remains simple enough for high-throughput applications.

The data from our study highlight several inherent advantages associated with a qRT-PCR-based approach. A concept known as the Percentage Law predicts that the neutralization titer of an antibody solution would not be impacted by the input virus dose, assuming that the antibody is in vast molar excess relative to the virus during a neutralization reaction (an assumption that should generally hold true under most assay conditions) [24], [25], [26]. Indeed, qPCR-MN appears to be compliant with the Percentage Law, as predicted (Table 7). The dynamic range of qRT-PCR (in our case, at least 5 log_10_; Fig. 1) is the basis that allows a direct demonstration of compliance with the Percentage Law without the need to alter assay parameters other than input virus dose. A comparable demonstration would be difficult for ELISA-MN due to the limited dynamic range of ELISA. The ELISA-MN protocol emphasizes that the input virus dose needs to be a tightly controlled parameter (100 TCID_50_) [6], [7]. We interpret this to be a limitation of the ELISA methodology rather than a true violation of the Percentage Law for ELISA-MN. In any case, qPCR-MN is likely to be more robust to perturbations in input virus compared with ELISA-MN.

The sensitivity of qRT-PCR allows endpoint assessment for qPCR-MN at 6 hours post-infection compared with ∼18–22 hours for ELISA-MN. A shorter duration for an assay is desirable in itself. However, aside from logistics, there may be a theoretical basis to prefer an endpoint assessment at 6 hours. Currently available neutralization assays for influenza virus, including ELISA-MN, are associated with a significant degree of intra- and inter-laboratory variability [11], [12], [13]; thus, a need exists for improvement in assay performance in terms of precision. An assessment at 6 hours, allowed by qPCR-MN, likely occurs within the initial round of virus infection. In contrast, an assessment at 18–22 hours, typical for ELISA-MN, likely occurs following multiple cycles of virus replication, particularly in the presence of exogenous trypsin. In this sense, in terms of analysis and data interpretation, qPCR-MN is similar to *bona fide* single-cycle assays such as MN assays using pseudotype reporter viruses [27], [28], [29], [30], [31], [32] (although it must be emphasized that qPCR-MN uses replication-competent viruses, and thus for work with highly pathogenic strains requiring stringent bio-containment, an alternative approach, such as the use of non-replicating pseudotype viruses, might be more appropriate). We have found a consistent, albeit modest (∼2-4-fold), distorting effect when virus neutralization proceeds through multiple cycles of virus replication. qPCR-MN results in the presence of trypsin are comparable with those in the absence of trypsin when the endpoint is assessed at 6 hours *vs*. 12 hours (Tables 4, 5, 6). At 22 hours in the presence of trypsin, 2–4 fold lower titers are observed compared with titers in the absence of trypsin (Tables 4, 5, 6). This phenomenon is also corroborated by our ELISA-MN results (Table 3). Overall, our data support the notion that a single-cycle assay of shorter duration simplifies the interpretation of virus neutralization results and avoids a source of bias that can potentially have a compounding effect on assay variability. It is notable that MN assays of longer duration, *i.e*., 3–7 days (based on assessment of CPE), are associated with greater variability compared with ELISA-MN of 18–22 hour duration [11], [12], [13]. This broader variability trend can be interpreted as additional justification for an expectation that variability might be further reduced by limiting assay duration to 6 hours.

Most laboratories now appear to perform ELISA-MN in the absence of exogenous trypsin [13]. ELISA-MN assessed at 22 hours post-infection in the absence of trypsin can be an approximation of a single-cycle assay (Table 3). However, strain-to-strain differences might be observed with respect to dependence on exogenous trypsin for infectivity in cell culture or other virus attributes, thereby resulting in an unintended variable that can influence assay results. Even in the absence of exogenous trypsin, the precision for qPCR-MN results obtained with SI/06 at 22 hours appears to be reduced compared with those obtained at 6 hours (Table 5). Recent studies suggest that trypsin can strongly inhibit interferon signaling in MDCK cells during infection with influenza virus through proteolytic degradation of secreted interferon [33], [34]. Thus, trypsin may facilitate influenza virus replication in a manner apart from its well-characterized effect on viral hemagglutinin proteolytic processing. The viral nonstructural protein 1 (NS1) counteracts interferon signaling [35], although again, there might be strain-to-strain differences in this function. It is plausible that cellular interferon response triggered by virus infection might be able to influence virus neutralization results. The issue of cellular innate immunity introduces a new level of complexity to the interpretation of virus neutralization results as well as another potential source of assay variability. qPCR-MN may circumvent this issue by allowing an assessment at 6 hours post-infection, prior to the full establishment of the anti-viral state induced by interferon [34]. For prudence, qPCR-MN can also be routinely performed in the presence of trypsin, because at 6 hours post-infection, trypsin does not appear to affect the extent of virus replication (Fig. 2) or qPCR-MN results (Tables 4–6).

Endpoint measurement by qRT-PCR might be more amenable to standardization across laboratories compared with ELISA. Reference reagents (purified RNA or SPR-derived cellular lysates) can be stored frozen and distributed in order to facilitate the establishment and optimization of qRT-PCR in a new laboratory. In contrast, a comparable reference antigen (influenza virus nucleoprotein) for ELISA would be less straightforward to generate.

The availability of the option to freeze qPCR-MN samples would also be useful in other circumstances. In case of a technical failure that occurs during ELISA for ELISA-MN, one would have to start again from the beginning with virus neutralization and cell infection. A comparable failure during qRT-PCR for qPCR-MN would simply necessitate a re-assessment of frozen samples.

For our qPCR-MN, preparation of sample cell lysates is accomplished manually in a few minutes. For downstream qRT-PCR, we routinely make use of an automated liquid handling system to prepare the reactions in 96-well plates. While not essential for our qPCR-based approach, automation facilitates throughput, improves data quality, and reduces cost (by allowing smaller assay volumes). While each individual step in ELISA is simple in nature, full automation might be more difficult to achieve because of the higher overall number of manipulations required.

It is important to note that ELISA-MN has a distinct advantage over qPCR-MN in terms of cost (∼$1 per qRT-PCR well, or ∼$100 per full 96-well qRT-PCR plate; driven mainly by the commercial reagents for sample preparation and one-step qRT-PCR). Thus far, we have largely focused on technical feasibility and logistical concerns aside from cost. However, the current cost associated with qPCR-MN might be acceptable for many applications. In addition, it might be possible to achieve further cost reduction by additional downscaling. Finally, market dynamics of supply/demand might, in time, result in lower reagent costs.

We conclude by commenting on the general applicability of the qRT-PCR-based approach. It may be possible to develop qRT-PCR-based neutralization assays for other RNA viruses in a relatively rapid manner. Specific reagents that are difficult or time-consuming to generate, such as antibodies or recombinant reporter constructs, are not required in an assay using qRT-PCR. As noted earlier [19], an endpoint assessment relying upon qRT-PCR might be particularly suited for viruses that grow slowly and/or induce limited CPE.
